# An overview of treatment options for urinary stones

**Published:** 2016

**Authors:** Hamid Shafi, Bobak Moazzami, Mohsen Pourghasem, Aliakbar Kasaeian

**Affiliations:** 1 Fatemeh Zahra Infertility and Reproductive Health Research Center, Babol University of Medical Sciences, Babol, Iran.; 2 Student Research Committee, Babol University of Medical Sciences, Babol, Iran.; 3 Cellular and Molecular Biology Research Center (CMBRC), Babol University of Medical Sciences, Babol, Iran

**Keywords:** Urolithiasis, Treatment modalities, Urinary tract stones

## Abstract

Urolithiasis has become a worldwide problem with the prevalence of the disease increasing over the past few decades. While various treatment modalities have evolved over the years, discrepancies exist regarding the clinical indications and the efficacy of each of these treatment options. In the present review, we aim to review the current treatment modalities for urinary tract stones to provide a better understanding on the therapeutic approaches as well as their clinical indications.

Urolithiasis is a worldwide problem that can affect all groups of ages and is one of the major sources of morbidity around the world. The prevalence of lifetime risk for urolithiasis has been increasing over time (1). It has been reported, that about 50% of patients with a history of urinary stones will have a recurrence of a second stone forming within the next 10 years (2- 4). In addition, other known causes of forming ureteric stones both in pediatric and adult populations include socioeconomic status, stone size, and location in urinary system, renal anatomy and abnormalities, climate and other factors, all of which have influence on the treatment outcome as well as the choice of intervention (5). The incidence of developing urinary calculi is about 0.5% per year in North America and Europe (6). Many dietary factors such as calcium and fluid intake have a major role in the formation of urinary stones (7-9). Epidemiological studies have shown DM and hypertension are also associated with stone formation (10-12). Over the last few decades, there have been great advancements in minimally invasive techniques. Currently, treatment options include extracorporeal shock wave lithotripsy (ESWL), percutaneous nephrolithotomy (PCNL), retrograde intrarenal surgery (RIRS) and laparoscopic ureterolithotomy. However, discrepancies exist among current clinical guidelines regarding the efficacy of these treatment options compared with each other. In the present review, we aimed to discuss the various treatment modalities for urinary tract stones to provide a better understanding on current treatment approaches. 

## Extracorporeal shock wave lithotripsy

The closed and controlled manipulation of the entire urinary tract defined as endourology was introduced during the late 1970s (13-15) extracorporeal shock wave lithotripsy (ESWL) was developed in Germany by Chaussy et al. and have revolutionized the treatment of both kidney and urinary lithiasis. Since its introduction in early 1980s, ESWL has become the first line treatment for renal stones, proximal stones, and midureteral stones because of its noninvasive nature, low costs, high efficiency of stone disintegration, less exposure of patients to anesthesia, shorter hospitalization and fewer complications (16-21).

ESWL is comprised of shattering forces produced by an external power source called lithotriptor, which produces high intensity and low frequency acoustic waves. All lithotripsy machines consist of 4 components: an energy source, a focusing system, a localization unit, and a coupling machine. The shock waves are concentrated directly onto renal or ureteral stone. The mechanism of fragmentation relies on cavitation, shear, and spalling (15). Cavitation is considered to be the most important force responsible for fragmentation of the stones into smaller pieces which can then be easily passed through the ureters (15). Also, for having a maximum efficacy on the outcome of the ESWL, several technical factors need to be taken into account, such as the energy level, type, size and location of the stone, presence of UTI, frequency of the pulses, endourologic skills and previous experience with ESWL (22-23).

According to AUA Ureteral Stone Clinical Guidelines (24), ESWL is considered as the first line treatment modality for calculi less than 1 cm. The success rate of ESWL decreases when stone is located in the lower pole (25). Lingeman et al. reported stone-free rates of approximately 30% for patients with lower pole calculi of 11–20 mm and 20% for patients with calculi >20 mm (25). 

Other factors related to renal anatomy such as hydronephrosis, stenosis of the ureteropelvic junction, horseshoe kidney and patient-related factors such as obesity, skin to stone distance and chronic renal disease, can also influence the result of ESWL (26-28).

Recent evidence has suggested the utility of ESWL for proximal ureteral stones which can be expanded to stones up to 15mm (29). Shafi et al. reported the success rate of 78.6% after 3 months of follow-up and also most of patients prefer ESWL over other procedures (29). Contraindications for ESWL treatment include pregnancy, uncontrolled urinary tract infections and obstruction, decompensated coagulopathy, arrhythmia, uncontrolled hypertension and renal artery or abdominal aortic aneurysm (24, 30). Almost in all cases, microscopic hematuria may occur but only about one third of patients will develop gross hematuria which are self-limiting in most cases and can be managed conservatively (31-33). Therefore, in summary, ESWL is a safe and effective method to treat stones in the urinary tract when proper indications are followed. Today, more than two decades after its implementation, the procedure is considered safe and while various side effects have been reported, most are rare and do not hamper the effectiveness of this technique. Preventive measures should be taken to minimize the frequency of these side effects.

## Percutaneous nephrolithotomy (PCNL)

Over the past two decades minimally invasive procedures have become widely accepted and have almost entirely replaced open surgery. Percutaneous nephrolithotomy (PCNL) has rapidly become a standard of care for the treatment of all stones greater than or equal to 2 cm (34). In 1976, Fernstrom and Johansson (35) were first to established PCNL as an accepted surgical procedure for extracting urinary calculi, whole or in fragments, under radiological control. However, of note, the risk of complications is higher than other endoscopic procedures, particularly if a surgeon is less experienced. The stone burden or composition will not affect the efficacy of PCNL which is the main advantage of this procedure (24). Pearle et al. reported that the stone free rate for stones smaller than 10 mm is 100 % of patients treated with PCNL, while only 63% for those treated with ESWL (36). Percutaneous removal of stones is currently indicated for patients with staghorn calculi, kidney stones greater than 2 cm, and lower pole stones greater than 1.0 cm (37). 

Contraindications to PCNL include uncorrected coagulopathy, urinary tract infections, inability to tolerate prone position especially in the case of respiratory compromise, and pregnancy. It is imperative to adequately treat any urinary tract infection prior to the procedure (38). Obtaining a proper access into the collecting systems is critical for safe and effective treatment. The procedure is performed using a posterior calyx usually in the upper or lower pole depending on the stone location and proximity of adjacent organs. Once the access to the collecting system is obtained, the tract to the renal pelvis is dilated using radiological assistance. Following these procedures, energy sources are used to break the stone in case intact removal of the stone is not feasible (39).

## Open Surgery

The surgical procedures for management of urolithiasis have dramatically changed over the past 3 decades. Back in 1980s, urologist routinely had to perform open surgery to extract stones from the urinary tract. Recent advances in endourological field, in the form of percutaneous nephrolithotomy (PCNL) and laparoscopy have resulted in a rapid decrease in the use of higher aggressive treatment approaches. Open surgery is needed in 1-5.4% of cases, according to the expertise worldwide (40-41). 

The current indications for open surgery according to European Association of Urology (EAU) (34) are as follows: complex stone burden, unsuccessful minimally invasive procedures such as ESWL or PCNL, comorbid medical diseases, morbid obesity, anatomical abnormalities (such as infundibular stenosis, PUJ obstruction, and stricture), skeletal deformity and nonfunctional kidney (nephrectomy) (42-44).

Therefore, while current emphasis is placed on minimally invasive stone treatments, open stone surgery maintains a small but significant role in the treatment of patients with renal and ureteral calculi. 

## Medical therapy

Medical expulsive therapy (MET) is a watchful waiting approach for treating urethral calculi and can be used successfully for a considerable number of patients (45-46). About 70% of ureteric stones are found in the lower third of the ureter at the time of presentation (4-5). Stones located in the distal portion of the ureter will have a successful spontaneous stone passage in about 50% of cases (45). The stone expulsion time depends on many factors consisting of stone size, location, and associated obstruction (47-49). Nevertheless, a watchful approach can result in a number of complications such as urinary tract infections, hydronephrosis, and colic events (50). 

According to American Urological Association (AUA) guidelines (51), the estimated spontaneous passage rate for stones <5mm is ranging from 71% to 98%, and for those measured 5 to 10 mm, stone passage rate is 25% to 53%. It has been estimated that the passage time for stones less than 2 mm is 8 days and for stones 4-6 mm, it may take 22 days for the passage of stones, respectively (47). However, it is not recommended to extend this conservative approach beyond 6 weeks, due to its potential risk of complications (51-52).

Alpha-adrenoreceptor antagonists (alpha-blockers), calcium channel blockers, and phosphodiesterase-5 (PDE5) inhibitors are believed to act by relaxing the ureteral smooth muscle to reduce ureteral contractions, inhibiting peristalsis and aiding in the elimination of stones (53-55, 27). This medical management also reduces the frequency of colic pain. The stimulation of the alpha1 adrenergic receptors in the ureter increases the force of ureteric contraction and the frequency of ureteric peristalsis. Blockade of alpha1 receptors inhibits basal tone, reduces peristaltic amplitude and frequency, and decreases intraluminal pressure while increasing the rate of fluid transport and the chances of stone expulsion. Alpha1A and alpha1D are the adrenergic receptor subtypes that are more densely expressed in the distal ureter (56).

Previously, three meta-analyses showed the utility of alpha-blocker therapy on the stone passage rate and these drugs had been shown to be the most effective for medical expulsion therapy (54). Tamsulosin (alpha-adrenergic blocker) which is used for treating patients with benign prostatic hypertrophy, have also been shown to have similar results in several trials. (57-59). 

While alpha-adrenergic blockers have been implicated as most effective therapies for the expulsion of urinary stones, other classes of drugs including thiazide and non-thiazide diuretics and alluporinol have shown to prevent the recurrence of nephrolithiasis (60). Among these classes of drugs, thiazides are the most widely used group of drugs in preventing calcium stones (60). Also, allopurinol shows to have a defined role in the prevention of calcium oxalate stones (61-62). Finally, nonthiazide diuretics such as indapamide emerged as an effective preventive strategy for calcium stone recurrences (63).

Consequently, while surgical modalities are still considered the mainstay of treatment for urolithiasis, medical expulsion therapy has recently emerged as an alternative treatment modality for the management of distal ureteric stones. 

In summary, treatment options for patients with urinary stones advanced significantly over the past few decades. Knowledge about each treatment modality including its advantages and adverse effects is necessary for physicians to be able to choose the best option for the patients. 
